# Using Wool Keratin as a Structural Biomaterial and Natural Mediator to Fabricate Biocompatible and Robust Bioelectronic Platforms

**DOI:** 10.1002/advs.202207400

**Published:** 2023-02-21

**Authors:** Shuihong Zhu, Qifan Zhou, Jia Yi, Yihua Xu, Chaoyu Fan, Changxu Lin, Jianyang Wu, Youhui Lin

**Affiliations:** ^1^ Department of Physics Research Institute for Biomimetics and Soft Matter Fujian Provincial Key Laboratory for Soft Functional Materials Research Xiamen University Xiamen 361005 P. R. China; ^2^ Wenzhou Institute University of Chinese Academy of Sciences Wenzhou 325001 P. R. China; ^3^ National Institute for Data Science in Health and Medicine Xiamen University Xiamen 361102 P. R. China

**Keywords:** natural mediator, robotic‐assisted manipulation, structural biomaterial, sustainable bioelectronic, wool keratin

## Abstract

The design and fabrication of biopolymer‐incorporated flexible electronics have attracted immense interest in healthcare systems, degradable implants, and electronic skin. However, the application of these soft bioelectronic devices is often hampered by their intrinsic drawbacks, such as poor stability, inferior scalability, and unsatisfactory durability. Herein, for the first time, using wool keratin (WK) as a structural biomaterial and natural mediator to fabricate soft bioelectronics is presented. Both theoretical and experimental studies reveal that the unique features of WK can endow carbon nanotubes (CNTs) with excellent water dispersibility, stability, and biocompatibility. Therefore, well‐dispersed and electroconductive bio‐inks can be prepared via a straightforward mixing process of WK and CNTs. The as‐obtained WK/CNTs inks can be directly exploited to design versatile and high‐performance bioelectronics, such as flexible circuits and electrocardiogram electrodes. More impressively, WK can also be a natural mediator to connect CNTs and polyacrylamide chains to fabricate a strain sensor with enhanced mechanical and electrical properties. With conformable and soft architectures, these WK‐derived sensing units can be further assembled into an integrated glove for real‐time gesture recognition and dexterous robot manipulations, suggesting the great potential of the WK/CNT composites for wearable artificial intelligence.

## Graphical abstract

Natural polymers show great potential in the research field of soft electronics. Wool keratin extracted from wool fibers can serve as a structural biomaterial and natural mediator for fabricating versatile, biocompatible, and robust bioelectronic platforms. The obtained platforms that can be applied in flexible circuits, gesture recognition, and robot manipulation are also being explored, providing a novel strategy to fabricate emerging soft electronics.

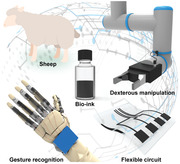

## 1 Introduction

The development of flexible and stretchable electronics has experienced a boom in the past decades owing to their emerging applications in personal healthcare, electronic skin, and robotic actuation.^[^ ^1^, ^2^, ^3^, ^4^, ^5^, ^6^, ^7^, ^8^, ^9^ ^]^ Natural polymers (such as silk, keratin, cellulose, and gelatin) offer an alternative option to construct sustainable soft electronics due to their intrinsic biocompatibility and environmental sustainability.^[^ ^10^, ^11^, ^12^, ^13^ ^]^ However, these current soft bioelectronic devices are generally limited by their poor electronic and mechanical properties, inferior scalable fabrication, and unsatisfactory durability.^[^ ^14^, ^15^, ^16^ ^]^ Biopolymer‐incorporated electroconductive inks have become promising candidate materials for fabricating flexible bioelectronic platforms within this framework.^[^ ^17^, ^18^, ^19^ ^]^ Particularly, carbon nanotubes (CNTs) have been widely regarded as a kind of ideal nanofillers for electroconductive inks owing to their unique superiorities.^[^ ^20^, ^21^, ^22^, ^23^, ^24^ ^]^ Nevertheless, the poor dispersibility of CNTs in most solvents hinders their practical application in flexible bioelectronics. Extensive efforts have been devoted to dispersing and stabilizing CNTs in water, such as surfactant addition, chemical modifications, and biological modifications.^[^ ^25^, ^26^ ^]^ But these strategies inevitably compromise the conductivity, chemical stability, mechanical properties, and biocompatibility of CNTs. Until now, only a few preliminary attempts and efforts have been made to use natural polymers to disperse CNTs.^[^ ^27^ ^–^ ^29^ ^]^

**Scheme 1:**
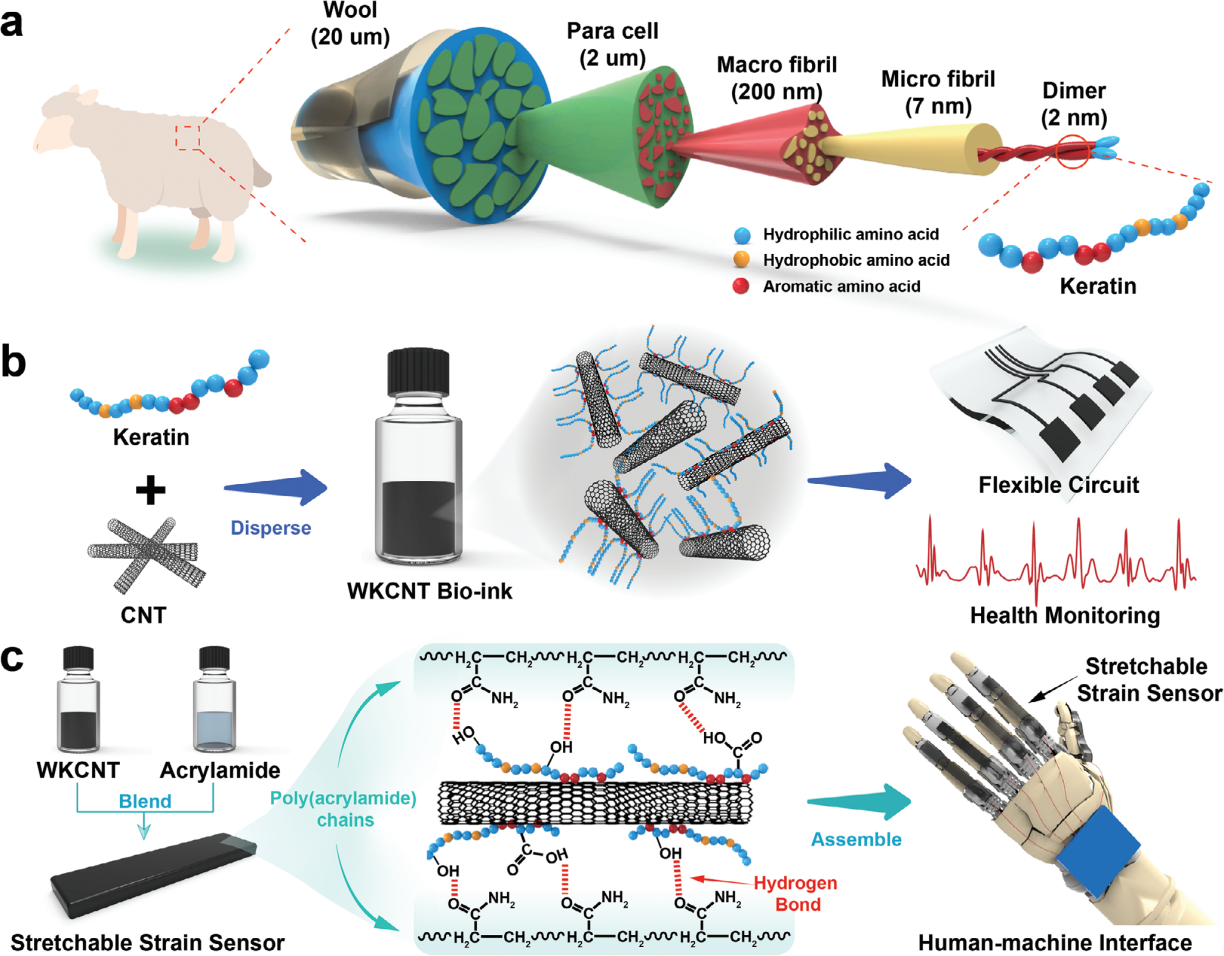
a) Schematic illustration of the structure of the wool fiber with different magnifications. b) Wool keratin‐derived electroconductive bio‐ink for a flexible circuit and health monitoring. c) Preparation of skin‐like stretchable strain sensors and applications for a human‐computer interface.

'Wool keratin (WK), a natural polymer extracted from wool fibers, has been widely reported in biomedical and pharmaceutical fields, resulting from its excellent hydrophilicity, controllable biodegradability, good biocompatibility, and programable processability.^[^ ^30^, ^31^, ^32^ ^]^ WK consists of a hierarchical structure and exhibits outstanding solubility in water due to a large number of hydrophilic amino acid residues (**Scheme** **1**a). Furthermore, the cell adhesion sequence, such as RGD peptide (R: arginine; G: glycine; D: aspartic acid) present in the WK peptide backbone, makes it a reliable choice for cell biology. Besides, WK contains a certain amount of aromatic amino acid residues, which can serve as binding sites on the surface of CNTs through strong *π*–*π* interactions, indicating the possibility of WK as a dispersant for preparing biocompatible conductive inks. More importantly, owing to the robust mechanical properties, WK also possesses excellent potential as a bio‐friendly structural biomaterial to enhance the performance of protein‐based bioelectronics.

Herein, for the first time, we propose a novel strategy to build biocompatible and robust bioelectronics using WK as a structural biomaterial and natural mediator (Scheme 1b). Both experimental and theoretical studies reveal that the unique features of WK can endow CNTs with excellent water dispersibility, stability, and biocompatibility. Well‐dispersed wool keratin‐CNTs (WKCNT) inks can be directly exploited to design versatile and high‐performance bioelectronics such as flexible circuits and electrocardiogram electrodes. By simply integrating with polyacrylamide, skin‐like stretchable strain sensors can be generated. More importantly, WK can also serve as a natural mediator to connect CNTs and polymer chains to improve the mechanical and electrical properties of strain sensors through hydrogen bonding interactions. These strain sensors were further assembled into a versatile electronic glove to collect somatosensory data for real‐time gesture recognition. Moreover, the wearable glove can be used as a creative human–machine interface for realizing effective and precise control of the robotic arm (Scheme 1c). As a proof‐of‐concept application, the hydrogel sensor modules and nanomaterials can be facilely synthesized through dexterous robot manipulation. The biocompatible and robust bioelectronic platforms developed here reveal their broad application prospects in home healthcare, wearable artificial intelligence, and advanced cybernetics.

## 2 Results and Discussion

### 2.1 Synthesis and Characterization of Electroconductive Bio‐Ink Dispersed by Wool Keratin

The regenerated WK was extracted from wool fibers by a moderate reduction method according to previously established protocols.^[^ ^33^, ^34^ ^]^ First, wool fibers were dissolved in an aqueous solution containing urea and sodium sulfide to break the disulfide bonds. Subsequently, the WK solution was obtained by removing the residual protein denaturants and reductants via dialysis. By analyzing the amino acid residue sequence of WK, the number of hydrophilic residues dominates the polypeptide backbone (Figure S1, Supporting Information). Additionally, WK also contains an appropriate amount of aromatic amino acid residues such as tyrosine (2.2%), tryptophan (0.5%), and phenylalanine (1.9%), which might provide the capability to obtain a strong *π*–*π* interaction with CNTs. Inspired by these unique features of WK, we reasoned that WK could act as a dispersant and stabilizer for constructing a new biocompatible aqueous ink. As expected, by simply shaking the mixed solution of WK and CNTs, WK exhibited intensive dispersibility and stability to CNTs instead of water (Video S1, Supporting Information). The CNTs powder could be further blended into the WK aqueous solution to yield a stable suspension through continuous stirring and ultrasonication. Remarkably, the aqueous WKCNT suspension showed high stability, allowing it to be stored for months without evident precipitation (Figure S2, Supporting Information). The unique formation of the WKCNT hybrid may lead to overcoming the massive van der Waals forces and strong *π*–*π* interactions between CNTs.

**Figure 1:**
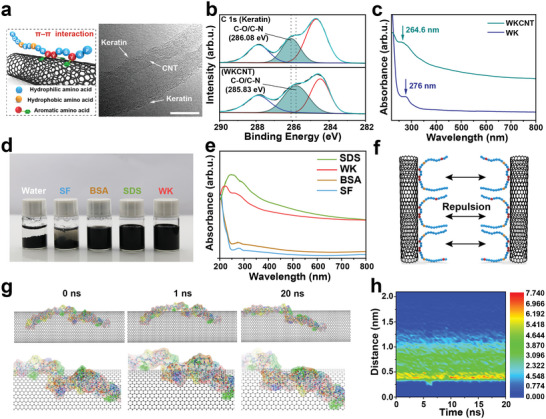
Characterization of the WKCNT electroconductive bio‐ink and molecular dynamic simulation of the interaction between CNTs and wool keratin. a) TEM image of wool keratin adsorbed on the surface of CNTs. The illustration on the left is a schematic diagram of adsorption. Scale bar = 10 nm. b) XPS spectra of C1s for keratin and WKCNT. c) UV–vis spectra of WKCNT bio‐ink and pristine wool keratin solution. d) Typical photographs of CNTs suspension with 5 mg mL^−1^ of different dispersants. e) UV–vis spectra of CNT dispersions with different dispersants. f) Schematic diagram of the mechanism of WKCNT bio‐ink maintaining stability. g) Snapshots of the system at 0, 1, and 20 ns, respectively. The bottom snapshots are enlarged views of the location of the tyrosine residue. h) Evolution of estimated density of the distance between the atoms of wool keratin and the surface of CNT.

To investigate the interaction of the WKCNT hybrid, transmission electron microscopy (TEM) was employed to directly observe the combination of WK and CNTs (**Figure** **1**a; Figure S3, Supporting Information). According to the TEM image, it could be clearly observed that WK was firmly adsorbed on the CNTs' surface. The illustration on the left in Figure 1a graphically shows the adsorption between WK and CNTs. Furthermore, X‐ray photoelectron spectroscopy (XPS) was utilized to explore the interaction mechanism of the WKCNT hybrid. As shown in Figure 1b, the C 1s XPS spectra of WK and WKCNT were displayed. Obviously, the peak of C—O/C—N of WKCNT shifted to the lower binding energy compared with the peak of C—O/C—N of WK (from 286.08 to 285.83 eV), suggesting that WK was successfully adsorbed on the CNTs surface.^[^ ^28^ ^]^ Additionally, the interaction of WK and CNTs was verified by Fourier transform infrared spectroscopy (FTIR), revealing the hydrogen bond formation between WK and CNTs (Figure S4, Supporting Information). Moreover, the Raman spectrum of WKCNT showed typical peaks of CNTs and WK (Figure S5, Supporting Information). To further confirm the dispersion of CNTs assisted by WK, UV–vis spectroscopy was employed (Figure 1c). The UV–vis spectrum of WK exhibited the typical characteristics of the protein. The peak at 276 nm, attributable to the aromatic amino acid residues in WK, slightly shifted to 264.6 nm in the WKCNT dispersion spectrum and showed a broader shape, demonstrating the interaction between the aromatic groups and the surface of CNTs. Several dispersants, such as proteins (silk fibroin (SF), bovine serum albumin (BSA)), and commercial surfactant sodium dodecyl sulfate (SDS), were selected to compare their ability to disperse CNTs (Figure 1d). The UV–vis spectrums of the dispersions with the same concentration of dispersants and CNTs were measured (Figure S6, Supporting Information). The UV–vis result revealed that the ability of WK to disperse CNTs was significantly better than SF and BSA and was comparable to that of SDS (Figure 1e). This result can be attributed to the unique structure of WK. With an inherent disorder of protein structure, WK generally cannot self‐assemble into a gel network or insoluble form and retains a high degree of freedom in the water. The aggregation of WKCNT hybrids will cause a decrease in the configuration entropy, while the energy of the system has hardly changed, thus unfavorable (Figure 1f).

Theoretically, molecular dynamics (MD) simulation was also taken to analyze the WKCNT hybrid interaction. A representative fragment of WK was chosen to investigate the adsorption of WK onto the CNTs' surface. Our simulation results showed that WK adsorbed to the surface of CNTs at the beginning of the simulation (0 ns) and tended to a stable state in the subsequent process (20 ns) (Figure 1g). Figure 1h shows a visualization of the distribution of keratin atoms over time. Experimental and theoretical results suggested that the aromatic amino acids in WK play an essential role in the adsorption process, endowing a strong *π*–*π* interaction between WK and CNTs. Collectively, the linear chain of WK protein with a large number of hydrophilic residues and an appropriate amount of aromatic amino acid makes it a promising candidate to disperse CNTs.

### 2.2 Biocompatibility and Scalability of the WKCNT Bio‐Ink

**Figure 2:**
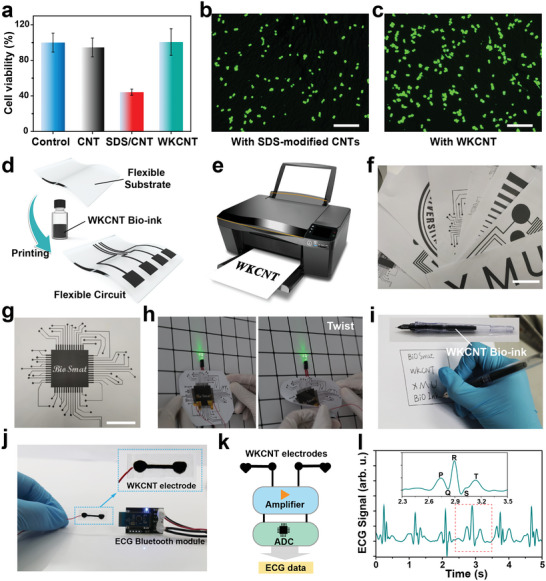
Biocompatibility and scalability of the WKCNT bio‐ink. a) Relative viability of RAW264.7 cells after incubation with 0.01 mg mL^−1^ pristine CNTs, SDS‐modified CNTs, and WKCNT. Typical fluorescence microscopy image of RAW264.7 cells after incubation for 48 h with b) SDS‐modified CNTs and c) WKCNT. Scale bar = 200 µm. d) Schematic diagram of WKCNT bio‐ink fabricated flexible circuit. e) The printing process of a commercial inkjet printer. f,g) Photograph of flexible circuit printed on paper by a commercial inkjet printer. Scale bar = 5 cm. h) The WKCNT‐based flexible circuit lights the LED light in the normal and twisted states. i) Direct writing with WKCNT bio‐ink. j) WKCNT‐based ECG electrode and Bluetooth acquisition module. k) Schematic diagram of the ECG system based on WKCNT electrodes. l) ECG signals collected by the WKCNT‐based ECG electrode. The inserted image shows the details of the ECG signal framed by the red dashed line.

Many previous reports have explored that WK is a bio‐friendly biomaterial due to its unique properties, such as RGD peptides.^[^ ^31^, ^32^ ^]^ To verify the biocompatibility of WKCNT, mouse leukemia cells of monocyte macrophage (RAW 264.7) were chosen as a model cell to perform cell incubation. The Petri dishes containing RAW 264.7 cells were loaded with 0.01 mg mL^−1^ pristine CNTs, SDS‐modified CNTs, and WKCNT, respectively, and incubated for 24 h. As shown in **Figure** **2**a, the cell viability of the WKCNT group was comparable to that of the control group. In contrast, the cell viability of SDS‐modified CNTs was significantly lower, indicating that CNTs dispersed with SDS were cytotoxic. After 48 h of coincubation, typical fluorescence microscopy images of the stained RAW 264.7 cells seeded in the SDS‐modified CNTs and WKCNT are presented in Figure 2b,c, suggesting WKCNT possessed better biocompatibility due to WK modifications. Moreover, the effects of different concentrations of pristine CNTs, SDS‐modified CNTs, and WKCNT on cell incubation were also verified (Figure S7, Supporting Information). Cytotoxicity testing results revealed that WK could significantly improve the biocompatibility of CNTs, demonstrating great potential for applications in cell engineering, implantable devices, and biomedicine. Next, the WKCNT‐based aqueous bio‐ink was successfully applied to prepare flexible and conductive circuits to verify its printability, conductivity, and scalability (Figure 2d). Through the I–V test on the WKCNT film fabricated by drop‐casting, the conductivity was calculated to be 39.36 ± 1.9 S cm^−1^, suggesting that the conductivity of the WKCNT film was comparable to other CNT inks (Figure S8, Supporting Information).^[^ ^27^ ^]^ By loading WKCNT bio‐ink directly into the cartridge of a commercial printer, various conductive circuits could be printed on flexible substrates such as paper and polyethylene (Figure 2e–g). The patterns obtained by printing could be used to light up the light‐emitting diode (LED), and the brightness of the LED had not changed after twisting, indicating that the circuits were flexible and conductive (Figure 2h). Other patterning techniques, such as direct writing (Figure 2i) and screen printing, have been verified for feasibility. In particular, the viscosity of WKCNT bio‐ink could be adjusted arbitrarily by adding thickening agents or diluting. Therefore, WKCNT bio‐ink can be employed as a promising material for fabricating printed flexible electronics. In terms of practical applications, electrocardiography (ECG) electrodes were developed by incorporating WKCNT bio‐ink with a simple stencil printing process. Excellent flexibility enabled the ECG electrodes to come into contact with the skin conformally, and good electrical conductivity allowed high‐quality ECG signals to be obtained (Figure 2j). After the collected physiological signals were processed by integrated circuits and programs (Figure 2k), the visualized ECG signals could be displayed to detect human health (Figure 2l). Unlike commercial gel‐based ECG electrodes that strongly rely on unstable gel materials, the WKCNT dry electrodes exhibited long‐term stability in the air and ease of use, providing a novel strategy to fabricate wearable health‐monitoring devices.

### 2.3 Skin‐Like Stretchable Strain Sensors Derived from WKCNT Electroconductive Ink

**Figure 3:**
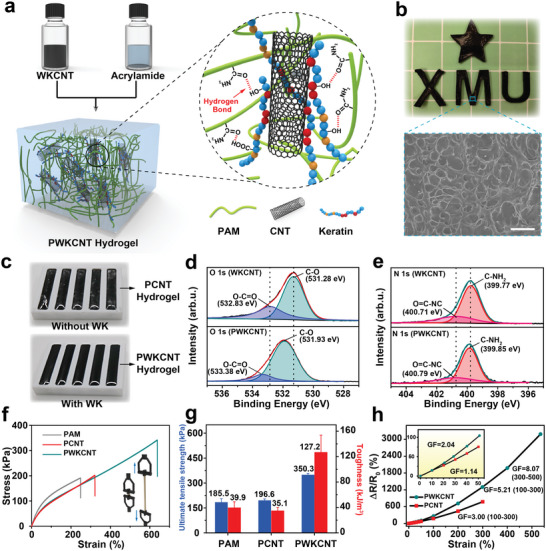
High‐performance PWKCNT hydrogel sensor modules. a) Schematic of WKCNT bio‐ink composited with acrylamide hydrogel. b) Photographs of PWKCNT hydrogels in various shapes and SEM image of PWKCNT hydrogel surface. Scale bar = 20 µm. c) Photographs of PCNT hydrogels without WK modification and PWKCNT hydrogels with WK modification. XPS spectra of d) O 1s and e) N 1s for WKCNT and PWKCNT. f) The tensile stress–strain curves of PAM, PCNT, and PWKCNT hydrogels. g) Ultimate tensile strength and toughness of PAM, PCNT, and PWKCNT hydrogels. h) The gauge factor of the PCNT and PWKCNT hydrogels.

Owing to their unusual mechanical behavior and biocompatibility, hydrogels have recently attracted intensive attention in various fields.^[^ ^35^, ^36^, ^37^ ^]^ Among them, polyacrylamide (PAM) has been widely reported in tissue engineering, soft actuators, and flexible wearable devices because of its unique mechanical properties and processability.^[^ ^38^, ^39^ ^]^ Despite the apparent advantages, the low conductivity hinders the prevalence of PAM hydrogels for flexible electronics. Adding conductive fillers (such as carbon nanomaterials, liquid metals, and conductive polymers) is an effective solution, but the dispersion of conductive fillers in water remains a challenge. Here, we employed WKCNT bio‐ink as the structurally enhanced and conductive filler to design high‐performance PAM/WKCNT (PWKCNT) hydrogels. Briefly, a stable WKCNT electroconductive ink was added to the pre‐prepared PAM aqueous solution containing reagents to initiate cross‐linking under continuous stirring. Subsequently, the PAM/WKCNT mixture was poured into a mold and left for several minutes to obtain the PWKCNT hydrogels. The detailed preparation can be found in Supporting Information. Owing to various functional groups on amino acid residues, such as amide, hydroxy, or carboxy, WK can act as a mediator between CNTs and hydrogel networks, which can form interactions (e.g., hydrogen bonds) between CNTs and polymer chains. As a result, the performance of as‐prepared PWKCNT hydrogels was improved significantly (**Figure** **3**a; Figure S9, Supporting Information). Benefiting from the dispersibility of WKCNT ink in water, CNTs could be uniformly dispersed in the hydrogel matrix without the assistance of any artificial additives (Figure 3b). As a comparison, the CNT‐water mixtures without WK modification aggregated and precipitated to the bottom of the mold during the cross‐linking reaction to form uneven PAM/CNT (PCNT) hydrogel (Figure 3c). To verify the possibility of WK as a mediator, XPS was also introduced to investigate the interaction mechanism between WKCNT and the hydrogel matrix. The high‐resolution O 1s and N 1s XPS spectra of WKCNT and PWKCNT are presented in Figure 3d,e. As seen in the O 1s XPS spectra, the C—O and O—C=O characteristic peaks of WKCNT underwent a significant shift from 531.28 to 531.93 eV and 532.83 to 533.38 eV, respectively, compared with PWKCNT.^[^ ^28^, ^40^ ^]^ Moreover, compared with PWKCNT, the C—NH_2_ and O=C—NC characteristic peaks of WKCNT were also slightly shifted from 399.77 to 399.85 eV and 400.71 to 400.79 eV, respectively, which might be attributed to the presence of C—O and O—C=O in WK dominating the formation of hydrogen bonds in the polymer network. As a result, the amide, hydroxy, or carboxy on WK can form hydrogen bond interactions with polymer chains after the dispersion of WKCNT in the PAM precursor.

**Figure 4:**
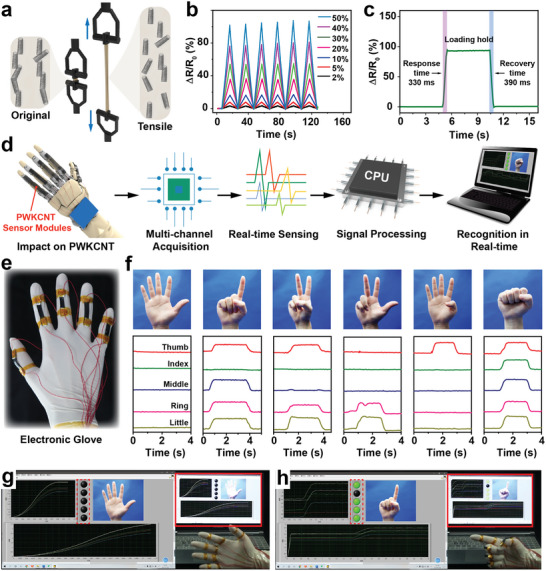
PWKCNT hydrogel modules for gesture‐to‐recognition translation. a) Mechanism of relative resistance change of PWKCNT hydrogels during tensile strain. b) Relative resistance changes of the PWKCNT sensing units at 2–50% tensile strain. c) The response and relaxation times of the PWKCNT sensing units under tensile strain. d) Schematic diagram of the hand motion signals from acquisition to recognition. e) Typical photograph of electronic gloves composed of PWKCNT hydrogel sensing units. f) Photographs and electrical signals corresponding to the sign language numbers 5, 1, 2, 3, 4, and 0, respectively. The real‐time screen display of sign language gestures g) number 5 and h) number 1.

Furthermore, tensile tests were implemented to investigate the effect of the homogeneous dispersion of CNTs in the polymer network. As expected, the mechanical properties of the strain sensors were significantly improved due to the assistance of WK (Figure 3f). The ultimate tensile strength of PWKCNT hydrogels compared to PAM hydrogels increased monotonically from 185.5 to 350.3 kPa, while the toughness also increased from 39.9 to 127.0 kJ m^−3^ (Figure 3g). However, the mechanical properties of PCNT hydrogels without WK modifications were essentially unchanged. In addition, the electrical performance of the strain sensors also improved dramatically with the WK regulation. As presented in Figure 3h, the GF values (namely gauge factor, GF = (Δ*R*/*R*_0_)/Δ*ε*, where Δ*R*/*R*_0_ represents relative resistance change, Δ*ε* stands for strain) of the homogeneously dispersed PWKCNT hydrogels were 2.04, 5.21, and 8.07 under tensile strains of 0–50%, 100–300%, and 300–500%, respectively. In contrast, the unevenly distributed PCNT hydrogels exhibited much lower GF values (1.14 and 3.00 at tensile strains of 0–50% and 100–300%, respectively). The mechanism of relative resistance changes of the PWKCNT sensing unit was explored in Supporting Information. The resistance changes under different deformations were captured further to investigate the strain‐sensing performance of the stretchable strain sensors (**Figure** **4**a). When tensile stress was applied to the PWKCNT hydrogel, the overlap among adjacent CNTs was decreased, resulting in an increment in relative resistance. Once the stress was withdrawn, the polymer network rapidly contracted to its initial state, restoring the relative resistance of PWKCNT hydrogel to its initial resistance. The results indicated that the PWKCNT sensors had a broad strain sensing range from 2% to 530% (Figure 4b; Figure S10b, Supporting Information), a quick response time of <390 ms (Figure 4c), and stability through thousands of cycles (Figure S10d, Supporting Information). Additional tension and compression tests can be found in Figures S10 and S11 (Supporting Information). The high sensitivity, fast response time, and reliable durability toward tensile and compress deformation enabled the PWKCNT hydrogels to monitor the full‐range human activities in real‐time. As proofs of concept, the high‐performance PWKCNT hydrogel modules were mounted on the muscles or joints to detect physical signals such as finger bending, finger tapping, and wrist pulse (Figure S12, Supporting Information).

In addition to the mechanical response, the skin‐like stretchable strain sensors exhibit thermosensation properties. As the temperature increased from 30 to 70 °C, the relative resistance of the PWKCNT sensor gradually decreased (Figure S13, Supporting Information). The temperature coefficient of resistance (TCR) was calculated to be −1.287% °C^−1^, which is comparable to the recently reported temperature sensors. Therefore, significant temperature fluctuations can affect the results of the electromechanical response. However, the temperature variation of the PWKCNT sensing unit during the test was negligible and thus not sufficient to cause a visibly negative impact on the electromechanical response, as verified by the tensile cycling tests performed at room temperature over a long period (Figure S10d, Supporting Information). Even when there is a wide range of temperature fluctuations, the electromechanical response can be adjusted by the dependence between relative resistance and temperature.

### 2.4 PWKCNT Hydrogel Modules for Gesture‐to‐Recognition Translation

Automatic human gesture recognition systems exert a significant influence on the investigation of intelligent human–machine interfaces (HMIs). However, conventional vision‐based approaches toward gesture recognition, which typically rely on images or videos, are easily affected by environmental disturbances such as visual noise and varying light conditions. In contrast, the classification of human gestures based on flexible and wearable bioelectronics, which can be integrated into the clothing or conformally attached to the human skin, allows a continuous, noninvasive, and real‐time translation of biometric signals without depending on the image quality.^[^ ^41^, ^42^ ^]^ Notably, monitoring human activities proved the capability of PWKCNT hydrogel to acquire sign language components. To accurately capture the somatosensory information, the PWKCNT hydrogel modules were assembled into a stretchable integrated glove, comfortably accommodating human hands and responding to finger motions. Since each hand gesture corresponds to a set of composite electrical signals, a gesture‐to‐recognition translation could be realized by combining the multi‐channel acquisition method. As illustrated schematically in Figure 4d, when multiple fingers moved simultaneously, the motion impacted on PWKCNT hydrogel modules was acquired by a multi‐channel device. At the same time, these analog signals were converted into digital signals by an analog‐to‐digital converter (ADC). Subsequently, the recognized gestures were displayed on the terminal in real‐time after signal processing. The electronic glove composed of PWKCNT hydrogel modules could accurately record and identify the numbers from 0 to 10 corresponding to different sign language hand gestures (Figure 4e,f; Figure S14, Supporting Information). The acquired electrical signals were analyzed by a program designed with LabVIEW. After a series of algorithmic processes, the corresponding gesture pictures were displayed on the computer screen in real‐time (Figure 4g,h). The output reliability of the gesture‐to‐recognition translation system was verified by repeating various hand gestures continuously in an orderly or disorderly manner (Video S2, Supporting Information). In addition, the lights framed by the red dotted line on the program interface correspond to the thumb, index finger, middle finger, ring finger, and little finger, respectively, from top to bottom. When a finger was bent, the motion information would light up the corresponding green light, which could be utilized to monitor the status of each finger visually. Therefore, this flexible bioelectronic platform will hopefully overcome the communication barrier between signers and non‐signers. We expect that the gesture‐to‐recognition translation system based on the PWKCNT hydrogel modules will have many practical applications, such as sign language translation for the deaf signer, mechanical manipulation, virtual reality, and patient rehabilitation training with hand injuries.

### 2.5 Robotically Assisted Gesture‐to‐Manipulation Translation System

**Figure 5:**
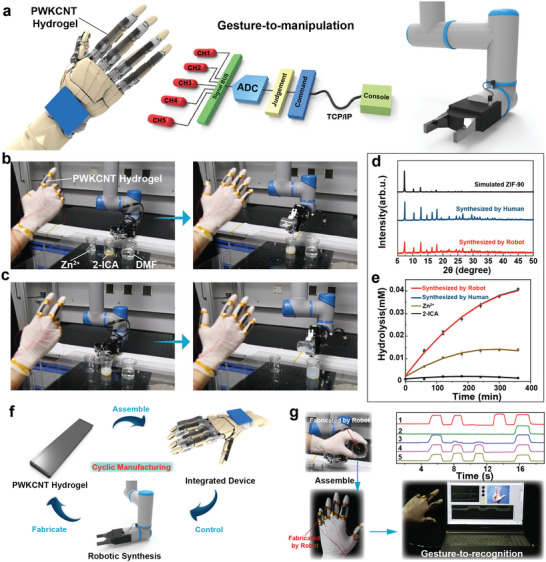
Robotic‐assisted gesture‐to‐manipulation translation system. a) Schematic diagram of PWKCNT‐based electronic glove controlling the robotic arm. b,c) Robotic‐assisted synthesis process of nanozyme ZIF‐90. d) XRD spectrum of ZIF‐90 nanozyme by the human, robot, and simulated ZIF‐90. e) Catalytic activity of ZIF‐90 synthesized by robot and human, Zn^2+^, and 2‐ICA. f) The fabrication of hydrogel sensor modules by the robotic‐assisted system. g) The PWKCNT hydrogel sensing units synthesized by the robotic arm are used for gesture‐to‐recognition.

Considering that the gesture‐to‐recognition translation system based on PWKCNT hydrogel can convert sign language components into distinguishable electrical signals, a robotically assisted gesture‐to‐manipulation translation system was proposed. As illustrated schematically in **Figure** **5**a, the dexterous manipulations were essentially based on three major procedures: Sign language components were first collected by multi‐channel acquisition and converted into digital signals by an ADC converter. Then the digital signals were converted into commands through quick judgment and transmitted to the console via Transmission Control Protocol/Internet Protocol (TCP/IP). Finally, the console controlled the robotic arm to complete programmable actions. Consequently, dexterous manipulations at various stations could be carried out by robots in the laboratory. Based on the robotic‐assisted gesture‐to‐manipulation translation system, the human hand could realize the flexible and scalable combination with a robotic arm, achieving communication between human and machine.

To demonstrate the capability of a robotic‐assisted platform for practical application, the synthesis of zeolitic imidazolate framework‐90 (ZIF‐90) nanozyme was chosen as a typical example. Phosphate hydrolase (OPH; EC 3.1.8.1), discovered in bacteria, exhibits excellent degradation activity toward organophosphorus nerve agents.^[^ ^43^ ^]^ The active site of OPH contains Zn^2+^ and first‐shell ligands (four histidines, carboxylated Lys 169, and Asp 301). ZIF‐90, by the assembly of Zn^2+^ and imidazole, could mimic the conformation of the active site in the natural enzyme OPH and then mimic the catalytic property of OPH (Figure S15a, Supporting Information). For instance, it could successfully hydrolyze the characteristic substrate of OPH, methyl parathion (MP), into the product p‐nitrophenol. Here, ZIF‐90 was synthesized by our robotic‐assisted manufacturing system. During the practical gesture‐to‐manipulation translation process, when the hand gesture was number 1, the motion signal was converted into command 1, and the robot arm was controlled to pour the Zn^2+^ solution into the imidazole‐2‐carboxaldehyde (2‐ICA) suspension. After 5 min of continuous stirring, hand gesture number 2 was executed to trigger the second step to pour the mixed suspension into the N, N‐dimethylformamide (DMF) solution (Figure 5b,c; Video S3, Supporting Information). The resulting nanoparticles were collected by centrifugation. And then, the morphology characteristics and crystal phase of ZIF‐90 were studied using transmission electron microscopy (TEM) and X‐ray diffraction (XRD). The robotically synthesized ZIF‐90 possessed similar 3D morphology (Figure S15b,c, Supporting Information) and characteristic peaks prepared by the standard simulated ZIF‐90 (Figure 5d). Additionally, the ability of ZIF‐90 nanozyme to mimic the OPH was measured by UV–vis spectrum monitoring the catalytic hydrolysis of the characteristic substrate MP to produce p‐nitrophenol (*λ*_max_ = 400 nm). As the catalytic reaction proceeded, the absorbance (*λ*_max_ = 400 nm) increased for p‐nitrophenol, and the solution color deepened (inset in Figure S15d, Supporting Information). Intriguingly, the ZIF‐90 nanozymes synthesized by the robotic arm and human exhibited almost the same OPH‐mimicking activity (Figure 5e). The results revealed the capability of the robotic‐assisted platform for nano‐synthesis as well as highlighted their promising potential, such as replacing humans to perform tedious and dangerous or, in some cases, high‐precision tasks without human interference.

In addition to the nanozyme synthesis, the robotic‐assisted system successfully implemented dexterous manipulations to produce hydrogel sensor modules. As depicted in Figure 5f, the PWKCNT hydrogel was assembled into an integrated device, and then the integrated device controlled the robotic synthesis to fabricate the PWKCNT hydrogel itself. The specific operation process is shown in Figure S16 and Video S4 (Supporting Information). The PWKCNT hydrogel synthesized by the robotic arm was used in the gesture‐to‐recognition translation system as proof of concept. The PWKCNT hydrogel fabricated by the robotic arm could continuously and stably complete the function of hand gesture recognition (Figure 5g; Video S5, Supporting Information). Furthermore, FTIR was performed to verify the long‐term stability of WK in the sensing unit (Figure S17, Supporting Information). A comparison between the freshly made PWKCNT hydrogel and the tested PWKCNT hydrogel left for 3 months showed that no significant degradation of WK occurred in the absence of protease, as well as indicating that the PWKCNT hydrogel can maintain its long‐term stability.

## 3 Conclusion

In summary, we developed WK‐incorporated biocompatible and robust bioelectronic platforms for human activities monitoring, sign language recognition, and robotic arm manipulation by using WK as a structural biomaterial and natural mediator. Experimental and theoretical studies revealed that the hydrophilic WK was absorbed onto the CNTs surface via non‐covalent interactions. As a result, the linear chain of WK protein with a large number of amino acid residues enabled the WKCNT bio‐ink to remain stable while enhancing the performance of the biopolymer‐based flexible devices. Flexible and conductive circuits could also be facilely prepared by combining electroconductive bio‐ink with printing techniques and exploited in human health monitoring, such as ECG signals. In addition, benefiting from the homogeneous dispersion of CNTs in water and the structural enhancements mediated by WK, the WKCNT bio‐ink could be further integrated with the hydrogel matrix to fabricate wearable and high‐performance PWKCNT sensing units. The obtained sensing units possessed high sensitivity (GF = 8.07 at 300–500% tensile strains), fast response time, and reliable durability toward tensile and compression deformation. The skin‐like stretchable strain sensors also exhibit thermosensation properties. Moreover, the PWKCNT hydrogel modules could be applied in sign language recognition and robotic arm manipulation based on a real‐time translation of electric signals to recognition and execution. The dexterous manipulations of a finger‐controlled robotic arm system were feasible to fabricate nanomaterials and hydrogel sensor modules. With their excellent sensitivity, broad scalability, and inexpensive fabrication, protein‐based devices can combine with wearable artificial intelligence. Therefore, we envision that such an attempt to build versatile, biocompatible, and robust bioelectronic platforms using wool keratin as a natural additive can advance the development of natural polymers in soft electronics.

## Conflict of Interest

The authors declare no conflict of interest.

## Supporting information

Supporting Information

Supplemental Video 1

Supplemental Video 2

Supplemental Video 3

Supplemental Video 4

Supplemental Video 5

## Data Availability

Research data are not shared.
