# Supplementary material for: Using Wool Keratin as a Structural Biomaterial and Natural Mediator to Fabricate Biocompatible and Robust Bioelectronic Platforms
Source: Adv Sci (Weinh). 2023 Feb 21;10(11):2207400. doi: 10.1002/advs.202207400 (PMC10104662; doi:10.1002/advs.202207400)
Supplement: Supplementary file 1 — Supporting Information [file ADVS-10-2207400-s006.pdf]

Supporting Information

Using Wool Keratin as a Structural Biomaterial and Natural Mediator to Fabricate Biocompatible and Robust Bioelectronic Platforms

*Shuihong Zhu, Qifan Zhou, Jia Yi, Yihua Xu, Chaoyu Fan, Changxu Lin, Jianyang Wu, and Youhui Lin**

S.H. Zhu, Q.F. Zhou, Y.H. Xu, C.Y. Fan, Prof. C.X. Lin, Prof. J.Y. Wu, and Prof. Y.H. Lin

Department of Physics, Research Institute for Biomimetics and Soft Matter, Fujian Provincial Key Laboratory for Soft Functional Materials Research, Xiamen University, Xiamen 361005, P. R. China

J. Yi

Wenzhou Institute, University of Chinese Academy of Sciences, Wenzhou, 325001 P. R. China

Prof. Y.H. Lin

National Institute for Data Science in Health and Medicine, Xiamen University, Xiamen 361102, P. R. China

Supplementary of experimental:

Materials. Sodium sulfide nonahydrate ($Na_2S \cdot 9H_2O$), urea (H_2NCONH_2), and sodium dodecyl sulfate (SDS) were purchased from Xilong Scientific Co. (Shantou, China). MWCNTs (TNM1) were purchased from Chengdu Organic Chemicals Co. Ltd. (Chengdu, China). According to the manufacturer, the MWCNTs have a purity of > 98%, a diameter of 5 - 15 nm, a length of 10 - 30 μm, and a specific surface area of 220 - 300 $m^2 \cdot g^{-1}$. Acrylamide (AM), ammonium persulfate

(APS), N, N′-methylene-bis-acrylamide (BIS), and N, N, N', N'-tetramethylethylenediamine (TEMED) were purchased from Macklin Co. Ltd. (China). Ultrapure water (18.2 MΩ; Millipore Co., USA) was used throughout the experiment.

Preparation of lyophilized powder of wool keratin. Wool keratin (WK) proteins were extracted and purified from wool fibers via the cleavage of disulfide bonds according to established protocols.[1, 2] The wool fibers were added to 50 mL of an aqueous solution containing sodium sulfide (6 g) and urea (24 g), and then placed in an oven at 60°C for 8 hours. The obtained wool keratin suspension was centrifuged at 9000 r/min three times to remove the insoluble residue. Then the resultant solution was put into a dialysis cassette (molecular weight cutoff, MWCO, 3500) for at least three days with frequent changes of deionized water. Finally, the lyophilized powder of wool keratin was obtained by lyophilizing the solution.

Preparation of WKCNT electroconductive bio-ink. The lyophilized wool keratin powder (0.1 g) was dissolved in deionized water (20 mL) to obtain a wool keratin solution. MWCNTs (0.1 g) were dissolved in the wool keratin solution under continuous mechanical stirring. Subsequently, the mixture was sonicated for 10 min under 750 W power using a probe sonicator (VCX750, SONICS & MATERIALS, INC.). Finally, the resultant suspension was centrifuged at 3500 rpm twice to remove undispersed MWCNTs.[3, 4]

Molecular dynamics simulation. The sequence of wool keratin was obtained from an online database.[5] A representative amino acid sequence of wool keratin containing 83 residues (SELRTMQNLEIELQSQLSMKASLENSLETKGRYCMQLAQIQEMIGSVEQLAQLRCEME QN QEYKILDVKTRLEQEIATYRLEG) was selected for molecular dynamics simulation. The structure of a (14,14) single-walled carbon nanotube was generated by a VMD plugin. The Wool keratin was placed nearby CNT with a minimum distance larger than 5.0 Å. The system was

simulated in a vacuum. The simulation box is set large enough to avoid periodic effects. All the simulations were performed by Gromacs (2019.6). The simulation was performed under the OPLS-AA forcefield, with parameters of CNT and protein obtained from the x2top and pdb2gmx modules, respectively. The system was first minimized to eliminate bad contacts. Subsequently, a constant temperature (NVT) ensemble was performed using the Nose-Hoover thermostat for 50 ns with 2 fs timestep. The data were analyzed with gmx-tools and Python scripts.

Cytotoxicity testing of WKCNT bio-ink. Mouse leukemia cells of monocyte-macrophage (RAW 264.7) were chosen as model cells for testing the biological compatibility of materials. RAW 264.7 cells were seeded into 96-well plates at a density of 1×10^4 cells per well in DMEM containing 10% FBS and 1% P/S and maintained at 37 °C in a 5% CO_2 atmosphere for 12 hours. Next, the cells were incubated with pristine CNTs, SDS-modified CNTs, and WKCNT for 24 hours. The original medium was aspirated, and the medium containing 10% Cell Counting Kit-8 was added and incubated for 1 hour. In addition, RAW 264.7 cells incubated with SDS-modified CNTs and WKCNT for 48 hours were stained by 0.5% (wt/vol) calcein for subsequent observation by fluorescence microscopy.

WKCNT electroconductive bio-ink for flexible circuits. The WKCNT electroconductive bio-ink was directly loaded into the cartridge (HP 803) and printed on paper using a commercial inkjet printer (HP 1112). Various patterns designed on the computer were printed on flexible copy paper and polyethylene. The flexible circuits were printed ten times to obtain a lower resistance, and then the LEDs were connected with wires. At the same time, the WKCNT electroconductive bio-ink could also be directly coated on the laser-cut mask to obtain flexible circuits. Furthermore, the WKCNT electroconductive bio-ink was put into a commercial pen instead of the original ink for handwriting.

Preparation of high-performance PWKCNT sensing unit. Due to the homogeneous dispersion in water, WKCNT bio-ink can be properly compounded with an acrylamide aqueous solution. The acrylamide precursor solution (15 mL) was composed of AM (6 g), APS (0.02g), and BIS (0.01 g). The WKCNT bio-ink (5 mL) was then added to the acrylamide precursor solution and kept stirring for five minutes, then TEMED (15 μL) was added, and finally, it was quickly poured into the mold to form PWKCNT hydrogel modules. Similarly, the PCNT hydrogels were obtained by adding MWCNTs (0.025 g) to the acrylamide precursor solution (20 mL) under continuous stirring. Obviously, during the PCNT hydrogels gelation process, MWCNTs aggregated and settled to the bottom of the mold without the assistance of WK by regulating interfacial interaction.

The mechanism of relative resistance changes of PWKCNT sensing unit. The sensing unit is regarded as a regular cylinder. When no external force is applied, the resistance (R) is given in Eq. 1:

$$R = \frac{\mathrm{\rho l}}{\mathrm{A}} \quad (1)$$

where ρ represents resistivity, l stands for resistance length, and A represents the cross-sectional area. When the stress deforms the sensing unit, ρ, l, and A change with the deformation. These parameters were used to obtain Eq. 2:

$$\frac{dR}{R} = \frac{\mathrm{d\rho}}{\mathrm{\rho}} + \frac{dl}{l} - 2\frac{dr}{r} \quad (2)$$

where r represents the radius of the cylinder. Eq. 3 and Eq. 4 can be obtained from the dependence between axial strain and radial strain:

$$\frac{dr}{r} = -\mathrm{\mu}\frac{\mathrm{dl}}{\mathrm{l}} \quad (3)$$

$$\frac{dl}{l} = \frac{\mathrm{\sigma}}{\lambda} \quad (4)$$

where μ represents Poisson's ratio, σ stands for stress, and λ represents elastic modulus.

Therefore, the relative change in resistance due to deformation can be obtained, as given in Eq. 5:

$$\frac{dR}{R} = \frac{\mathrm{d}\rho}{\rho} + (1 + 2\mu)\frac{\sigma}{\lambda} \tag{5}$$

According to Eq. 5, the sensor performance can be determined by three parameters, including resistivity (ρ), Poisson's ratio (μ), and elastic modulus (λ), which depend on the properties of the material.[6, 7]

Thermal-responsive performance of PWKCNT sensing unit. According to the previously reported temperature sensor,[4] the temperature coefficient of resistance (TCR) was obtained as follows:

$$TCR = \frac{1}{R_0} \cdot \frac{\Delta R}{\Delta T} \tag{6}$$

where R_0 is the initial resistance of the PWKCNT sensing unit at 30 °C, ΔR is the resistance change, and ΔT is the temperature change. As illustrated in Figure S13a, the TCR value was calculated to be -1.287% $°C^{-1}$. The resistance of PWKCNT hydrogel decreased with increasing temperature, which was attributed to the variable-range hopping transport mechanism.[8] The heating accelerates the mobility of the charge carriers, leading to an increase in electrical conductivity. The cyclically altered hot and cold temperatures were used to test the thermosensation properties, indicating high repeatability and stability of the PWKCNT sensing unit.

Construction of gesture-to-recognition translation system based on PWKCNT sensing units. The PWKCNT sensor unit was fixed on the finger joints with polyimide tape. Each sensing unit was connected to a resistor with matching resistance and then connected to a DC power supply in parallel. The five channels of the multi-channel acquisition were respectively connected to the

PWKCNT sensor units to monitor the state of the five fingers. The acquired electrical signals were analyzed by a program designed with LabVIEW. We set a threshold to judge the electrical signal obtained by each finger. If it exceeds the threshold, it means that the finger is in bending motion, and the green light on the interface will be lit; otherwise, it will not be lit. In addition, we entered the linkage of each gesture image and the corresponding electrical signal into the program in advance. When a specific gesture was executed, this group of electrical signals matched the electrical signal corresponding to the image of the gesture, and the program displayed this image to complete the recognition process of the gesture.

Robotic-assisted gesture-to-manipulation translation system. The electrical signal obtained by the PWKCNT sensing unit can be converted into the action command of the robotic arm (Siasun GCR5) through a program. When the sign language gesture was number 1, the electrical signal was converted into command 1 and transmitted to the console to drive the robotic arm to complete the first action. That is, the robot arm poured the WK solution into the beaker containing CNTs and continued mechanical stirring. Similarly, other movements of the robotic arm could be controlled by gestures. When the sign language gesture was number 2, the WKCNT bio-ink was mixed into the PAM precursor solution. Finally, when the sign language gesture was number 3, the mixture of WKCNT and acrylamide was poured into a beaker containing TEMED and then continuously stirred for five minutes to obtain the PWKCNT hydrogel. Therefore, we have realized the construction of a system that uses gestures to control the actions of the robotic arm.

Characterization. The adsorption of WK on the surface of CNTs was investigated by transmission electron microscopy (JEM 2100F, JEOL). XPS spectra were obtained using ESCALAB 250XIþ from Thermo Fisher Scientific. The UV-Vis spectrums were recorded by a

UV-Vis spectrophotometer (Lambda 750, PerkinElmer, Inc.). FTIR spectroscopy was performed 64 scans with a resolution of 0.4 cm^{-1}, and the wavenumber ranged from 4000 - 1000 cm^{-1} for each measurement with a Nicolet IN10 spectrometer (Thermo Fisher Scientific, USA). The Raman spectra were investigated using a Raman spectroscope (WITec alpha 300RA). X-ray diffraction (XRD, Panalytical X'pert Pro) with a Cu Kα source was employed to characterize the phase structure. The mechanical properties, including tensile and compressive deformation, were completed by Microtester 5948. The electrical signals were recorded by Precision LCR Meter (TH2829A) and output by the program designed with LabVIEW. The electrical signals of the electronic glove were collected by a multi-channel acquisition (USB 6259, NI).

MS**F**N**F**CLPNLS**F**RSSCSSRPCVPSSCCGTTLPGACNIPANVGSCN**WF**CEGS**F**N
GNEKETMQ**F**LNDRLAS**Y**LEKVRQLERENAELESRILERSQQQEPLVCPN**Y**QS
YFRTIEELQQKILCAKSENARLVVQID**W**NAKLAADD**F**RTK**Y**ETELGLRQLVE
SDINGLRRILDELTLCKSDLEAQVESLKEELICLKSNHEEEVNTLRSQLGDRLN
VEVDAAPTVDLNRVLNETRAQ**Y**EALVETNRRDVEE**WY**IRQTEELNKQVLSS
SEQLQSCQTEIIELRRTVNALEVELQAQHNLRDSLENTLTETEAR**Y**SCQLNQV
QSLISNVESQLAEIRGDLERQNQE**Y**QVLLDVRARLECEINTDRGLLDSEDCKL
PCNPCATTNACGKTITPCISSPCAPAAPCTPCVPRSRCGPCNS**Y**VR

Figure S1. The sequence of a typical kind of wool keratin. Blue, yellow, and red letters represent hydrophilic, hydrophobic, and aromatic residues, respectively.

CNT.

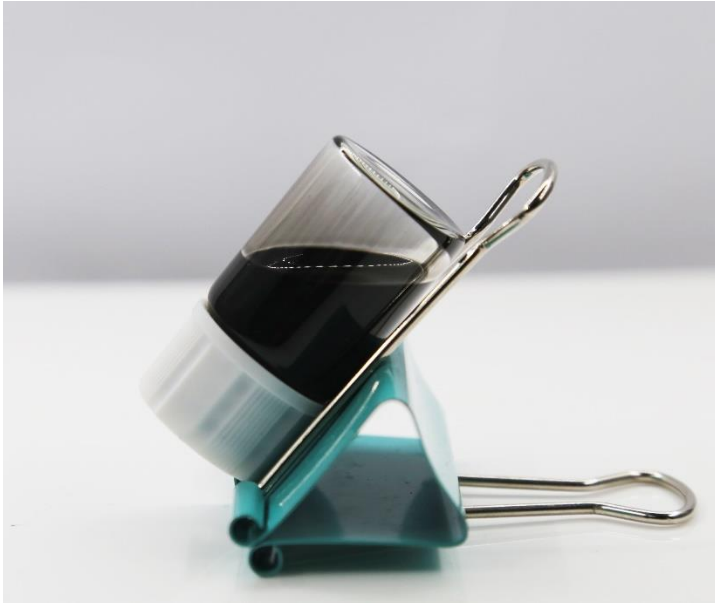

Figure S2. Optical photographs of WKCNT electroconductive bio-ink after one month after the preparation.

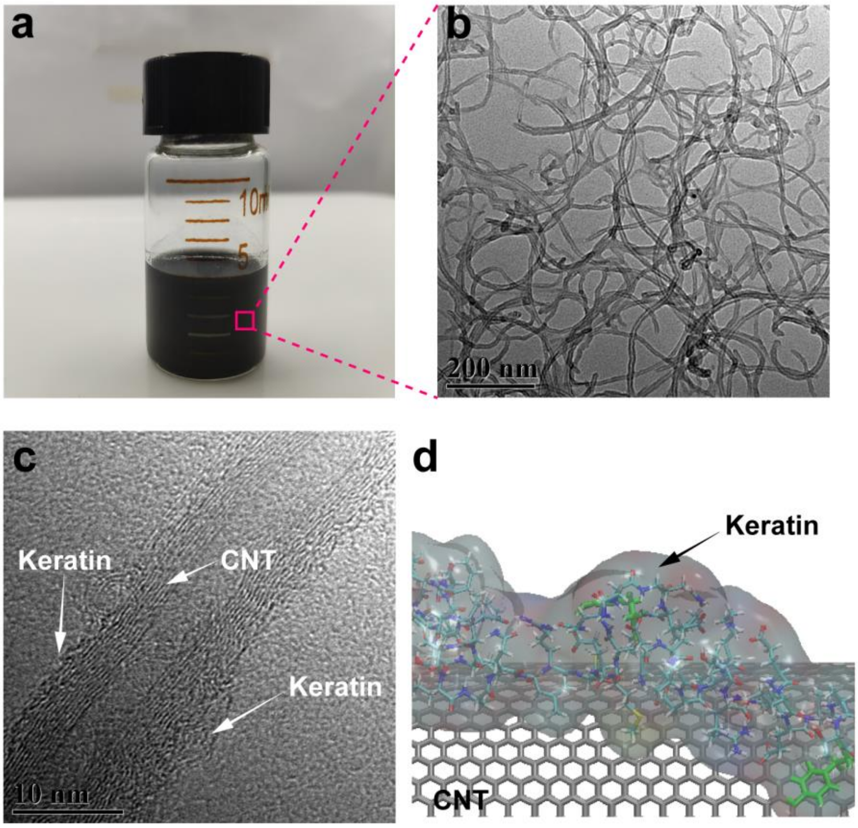

Figure S3. a) Optical photographs of WKCNT electroconductive bio-ink. b) TEM image of homogeneously dispersed WKCNT ink. c) Magnified TEM image of b). d) Schematic diagram of adsorption between WK and CNT.

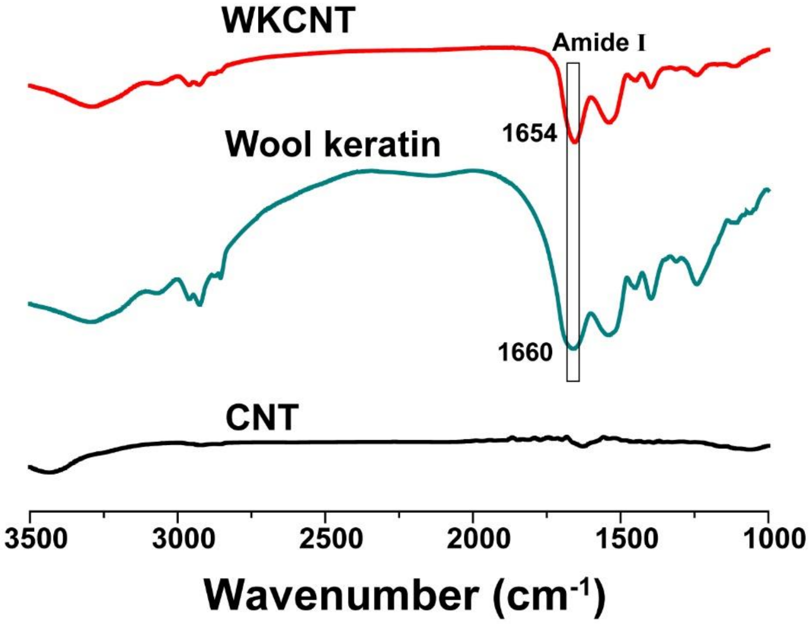

Figure S4. FTIR spectra of CNT, wool keratin, and WKCNT. The amide I peak of WKCNT slightly shifted from 1660 to 1654cm^{-1} compared with pristine wool keratin, suggesting hydrogen bond formation between wool keratin and CNTs.

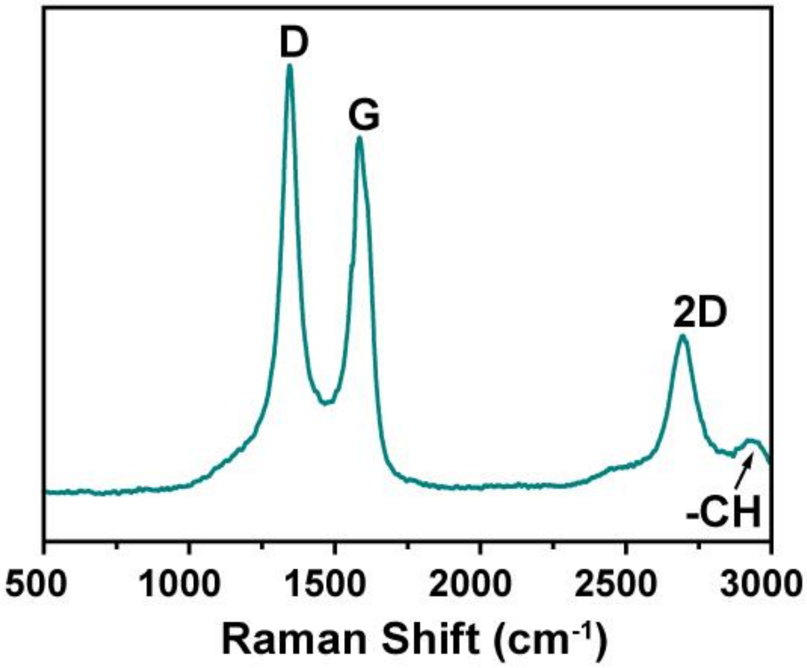

Figure S5. Raman spectrum of WKCNT film. Typical peaks of CNTs could be observed (D, G, and 2D). A peak at 2919 cm^{-1} was assigned as the wool keratin's stretching mode of C-H bonds.

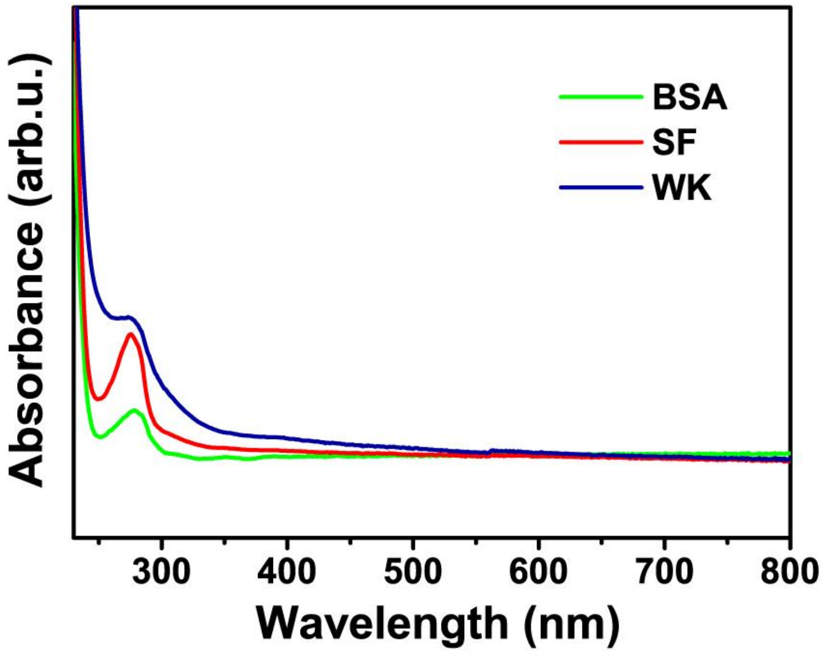

Figure S6. UV-Vis spectrums of the protein dispersants, including BSA, SF, and WK.

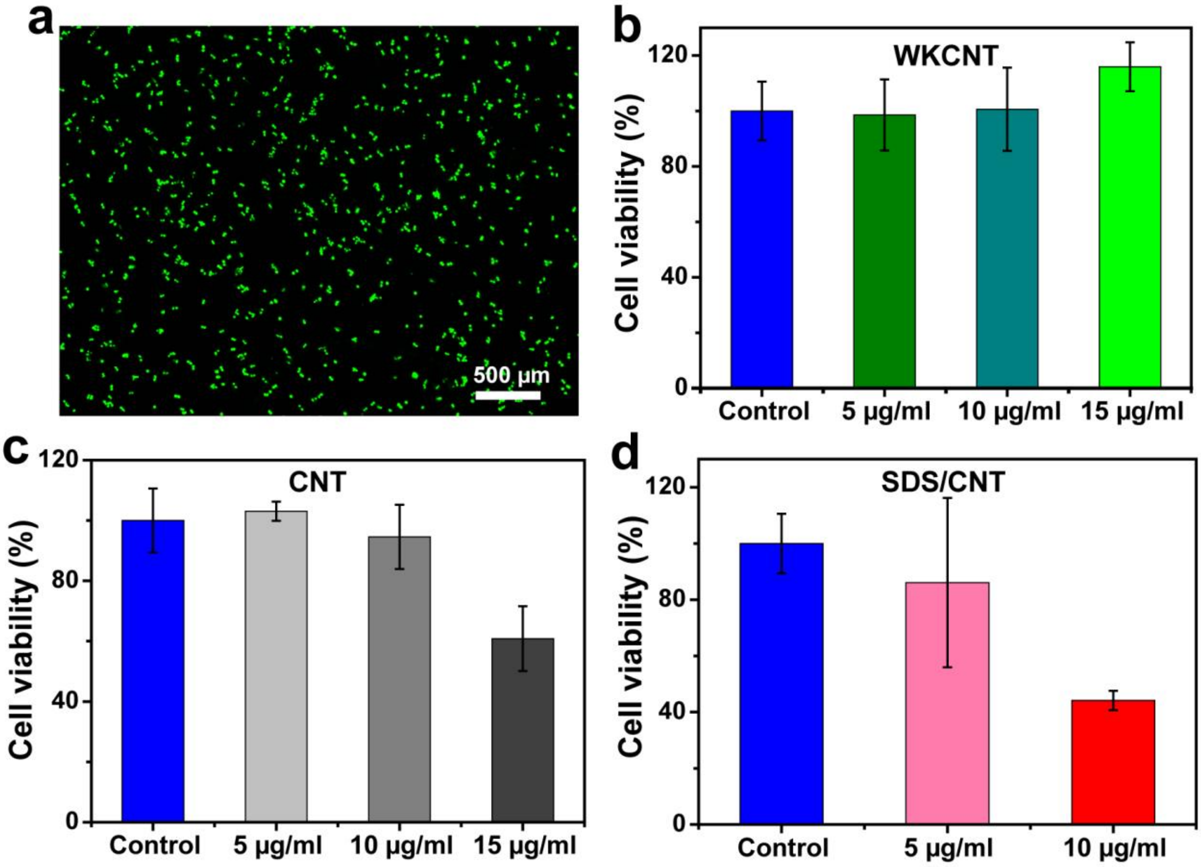

Figure S7. a) Fluorescence image of RAW264.7 cells after incubation for 48 hours with WKCNT. Relative viability of RAW264.7 cells incubation with different concentrations of b) WKCNT, c) pristine CNTs, d) SDS-modified CNTs.

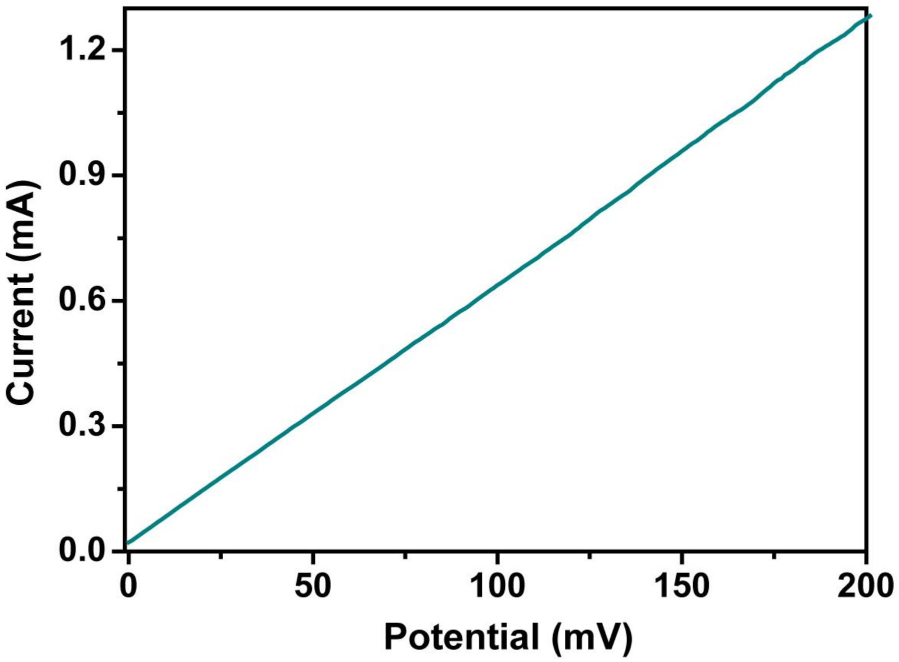

Figure S8. The I-V test on the WKCNT film fabricated by drop-casting.

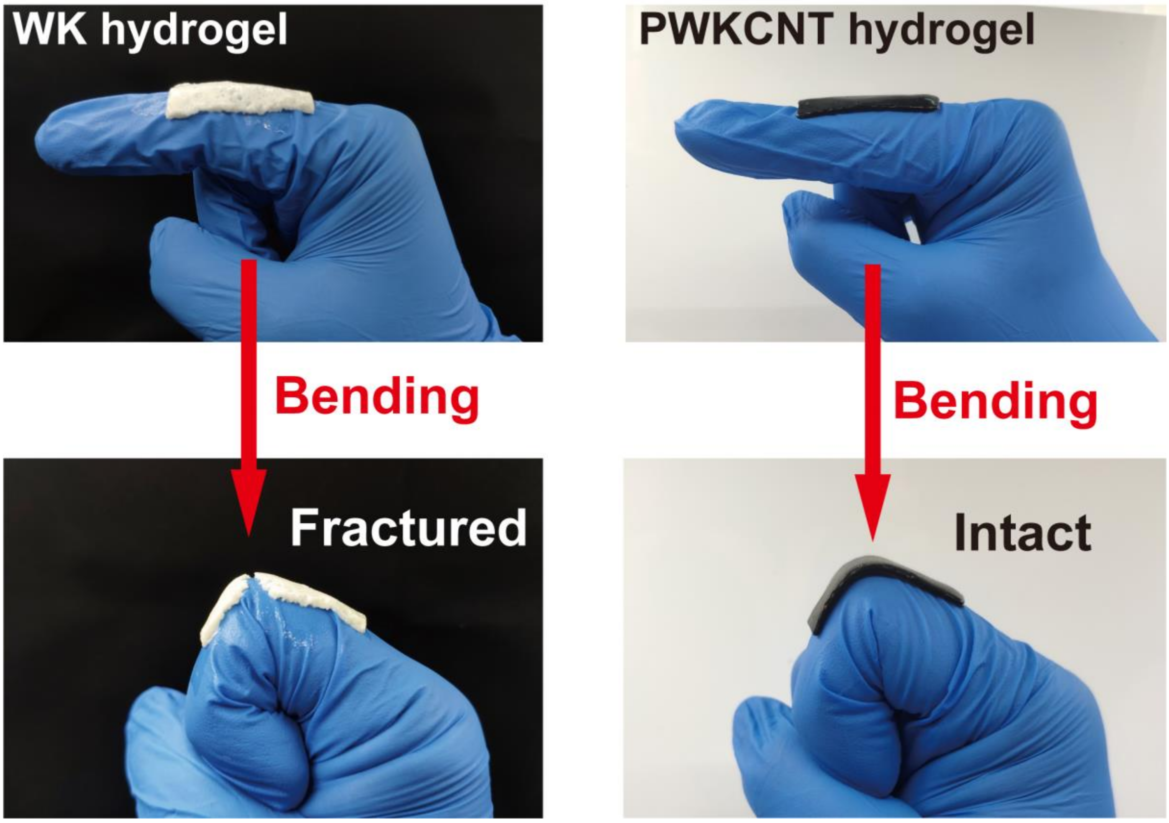

Figure S9. Comparison of mechanical properties of WK hydrogel and PWKCNT composite hydrogel.

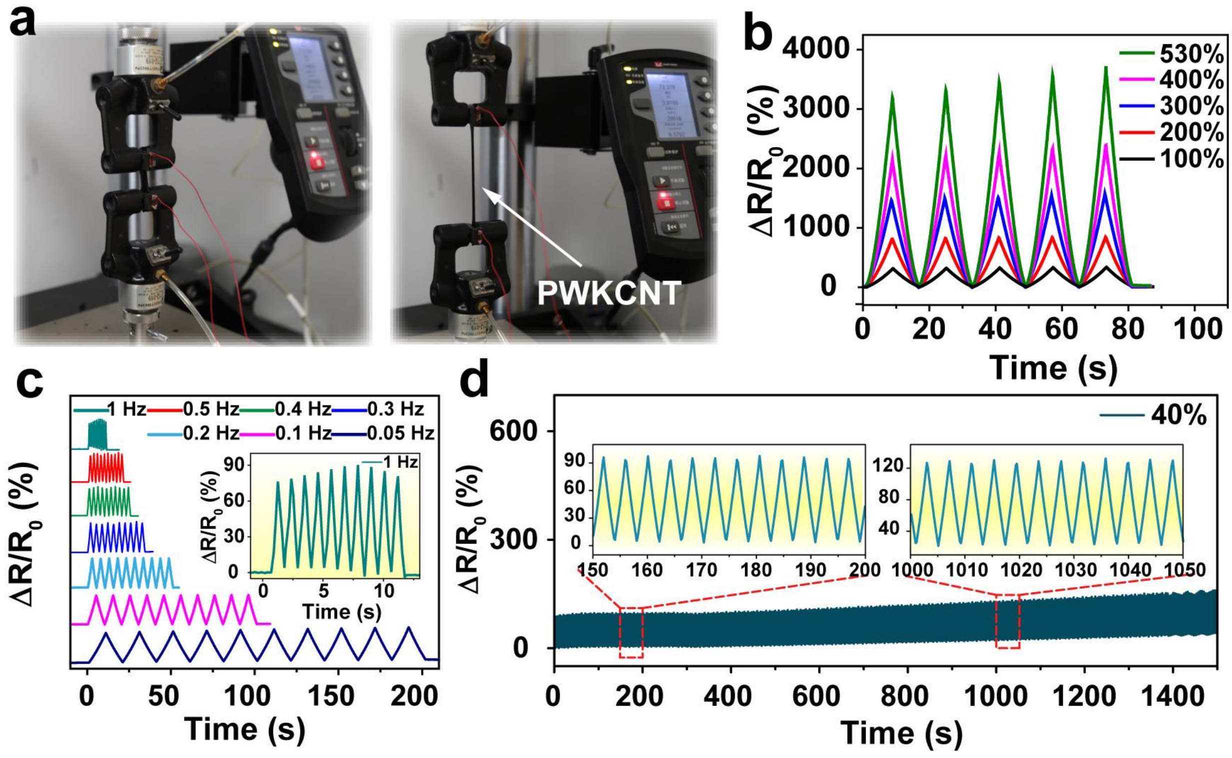

Figure S10. The electromechanical properties of PWKCNT sensing units under tension deformation. a) Optical photographs of the stretching process. b) Electromechanical properties of PWKCNT sensing units at 100 %-530 % tensile strain. c) Relative resistance changes of the PWKCNT sensing units at different frequencies under 40% tensile strain. d) The cyclic stability test of the PWKCNT sensing units under 40% tensile strain for 1500 seconds.

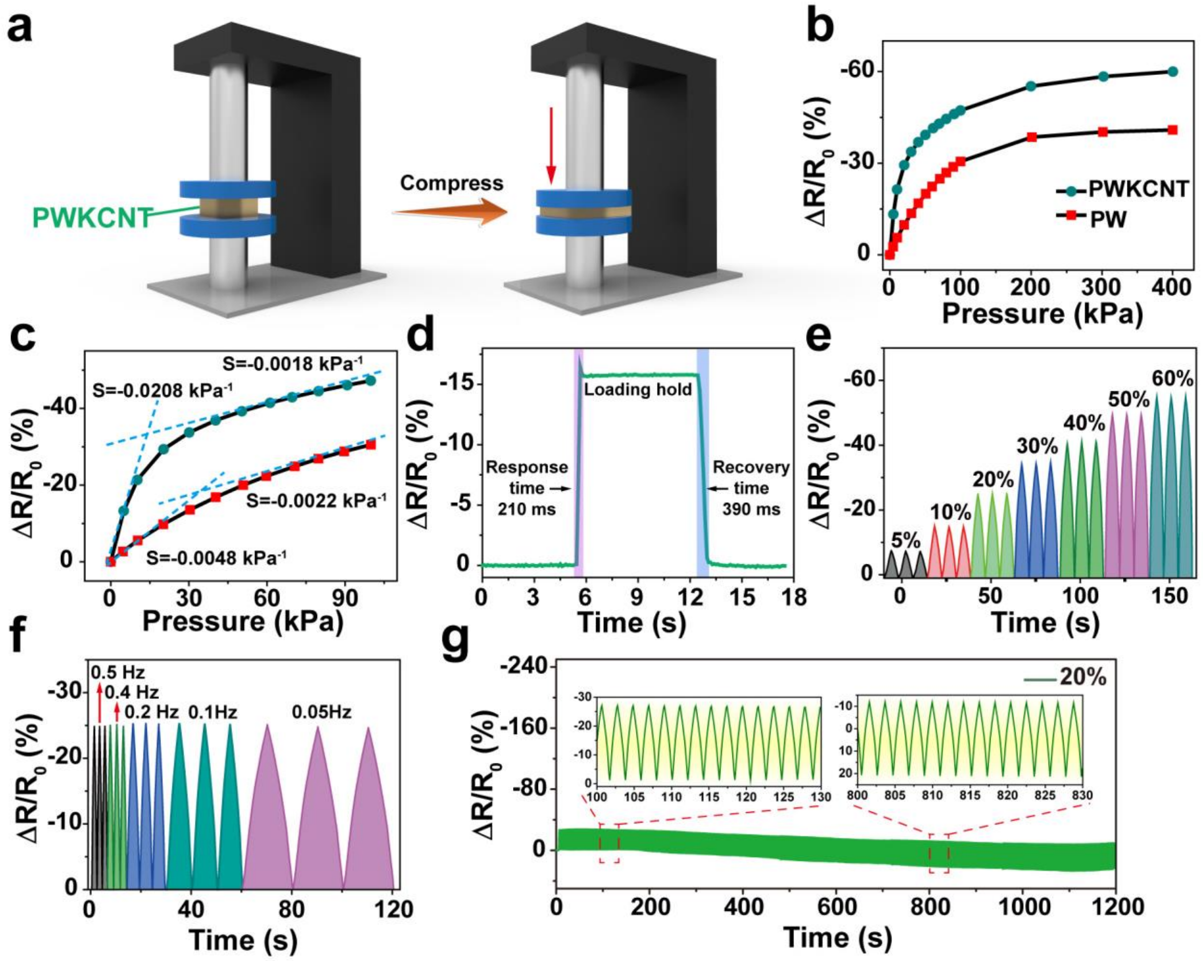

Figure S11. The electromechanical properties of PWKCNT sensing units under compression deformation. a) Schematic of the compression process. b-c) Sensitivity of the PWKCNT and PCNT hydrogels under different pressures. d) The response and relaxation times of the PWKCNT sensing units under compression deformation. e) Electromechanical properties of PWKCNT sensing units at 5 %-60 % compress strain. f) Relative resistance changes of the PWKCNT sensing units at different frequencies under 30% compress strain. g) The cyclic stability test of the PWKCNT sensing units under 20% compress strain for 1200 seconds.

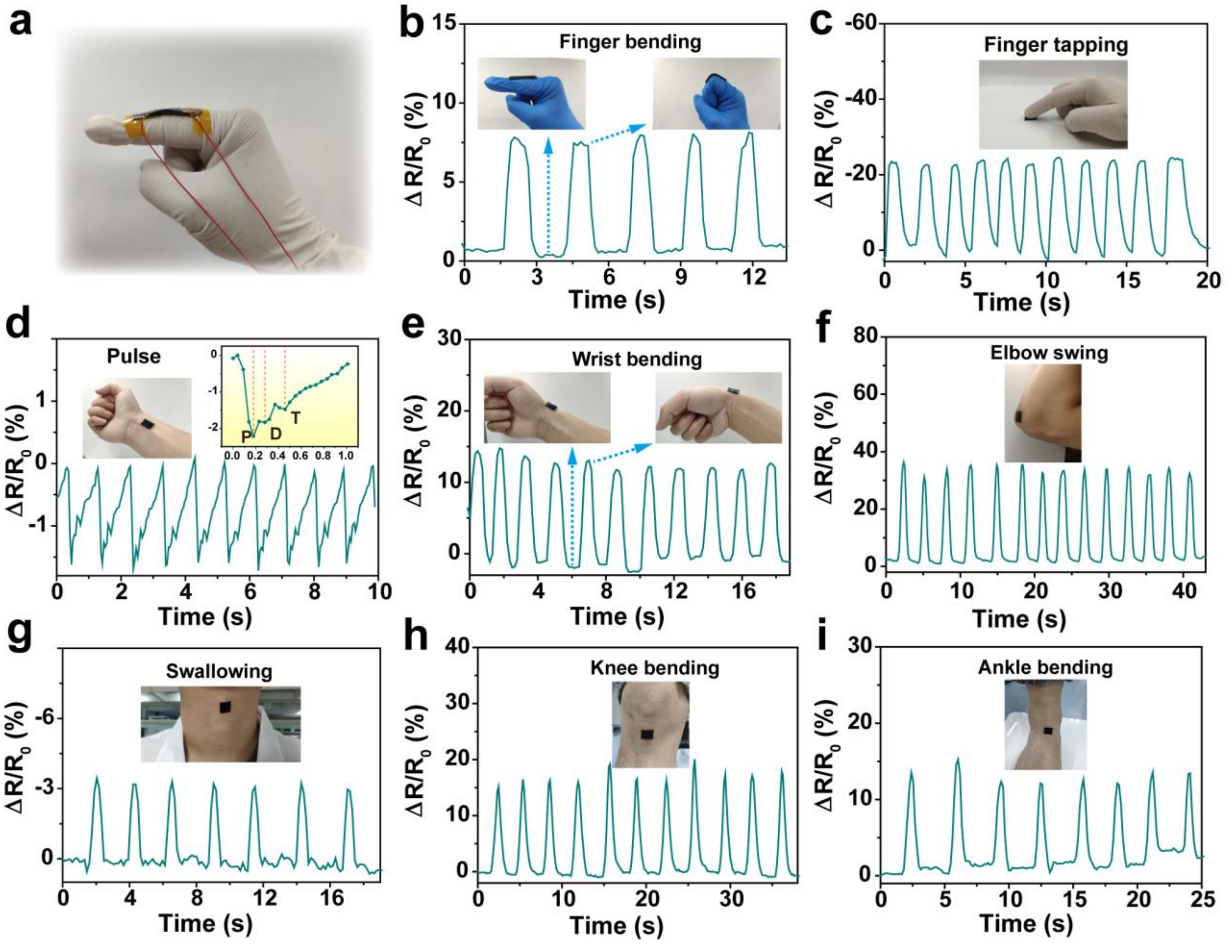

Figure S12. PWKCNT sensing units for Human activity monitoring. a) Optical photograph of PWKCNT sensing unit. Relative resistance changes of the PWKCNT sensing units at b) finger bending, c) finger tapping, d) wrist pulse, e) wrist bending, f) elbow swing, g) swallowing, h) Knee bending, and i) ankle bending.

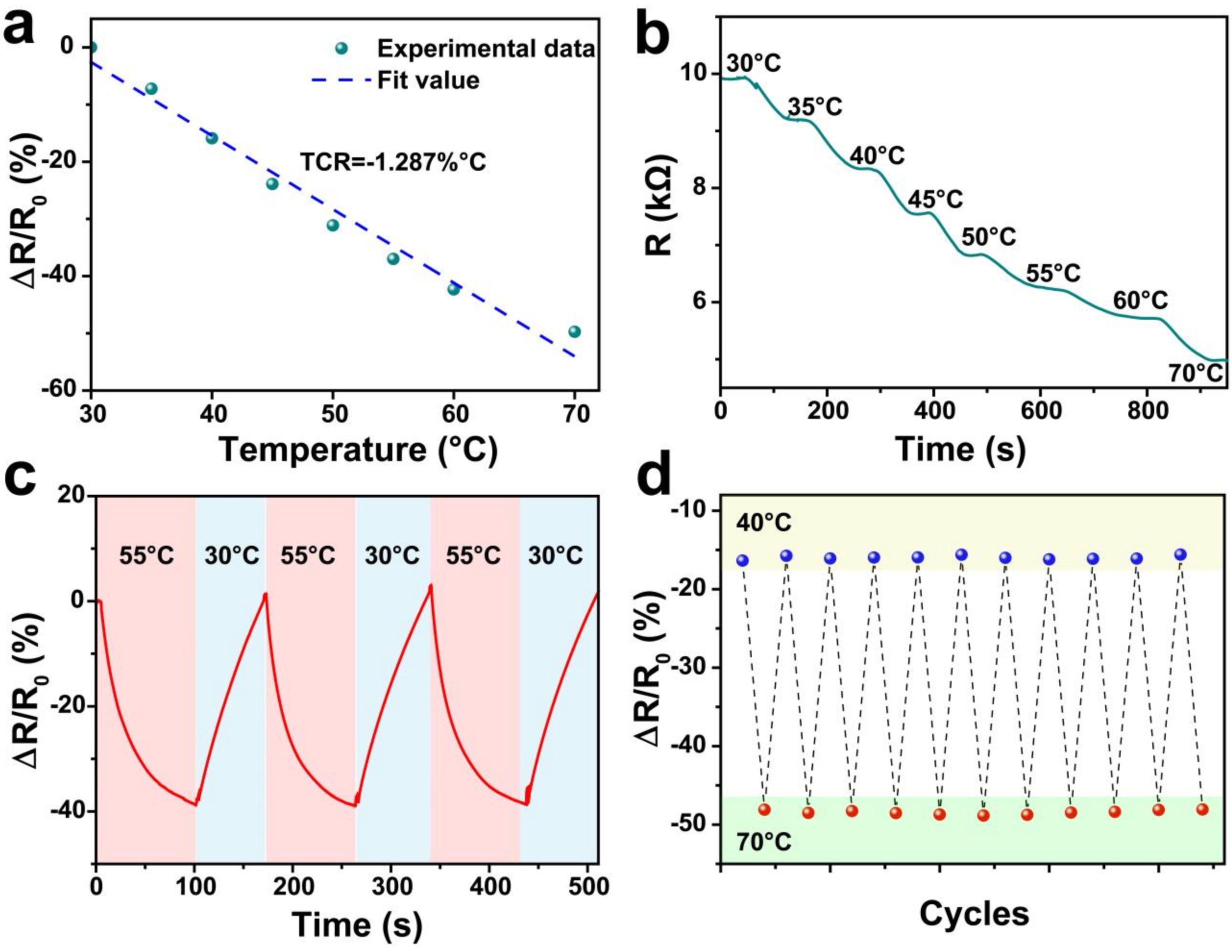

Figure S13. Thermal-responsive performance of PWKCNT hydrogels. a) The relative resistance of PWKCNT hydrogels changes with increasing temperature (from 30 to 70 °C). b) The resistance of PWKCNT hydrogel changes with increasing temperature. c) Real-time relative resistance changes of PWKCNT hydrogel when exposed to heat (55 °C) and cold (30 °C) sources. d) The ability of PWKCNT hydrogels to recognize temperature cycles during heating and cooling cycles between 40 °C and 70 °C.

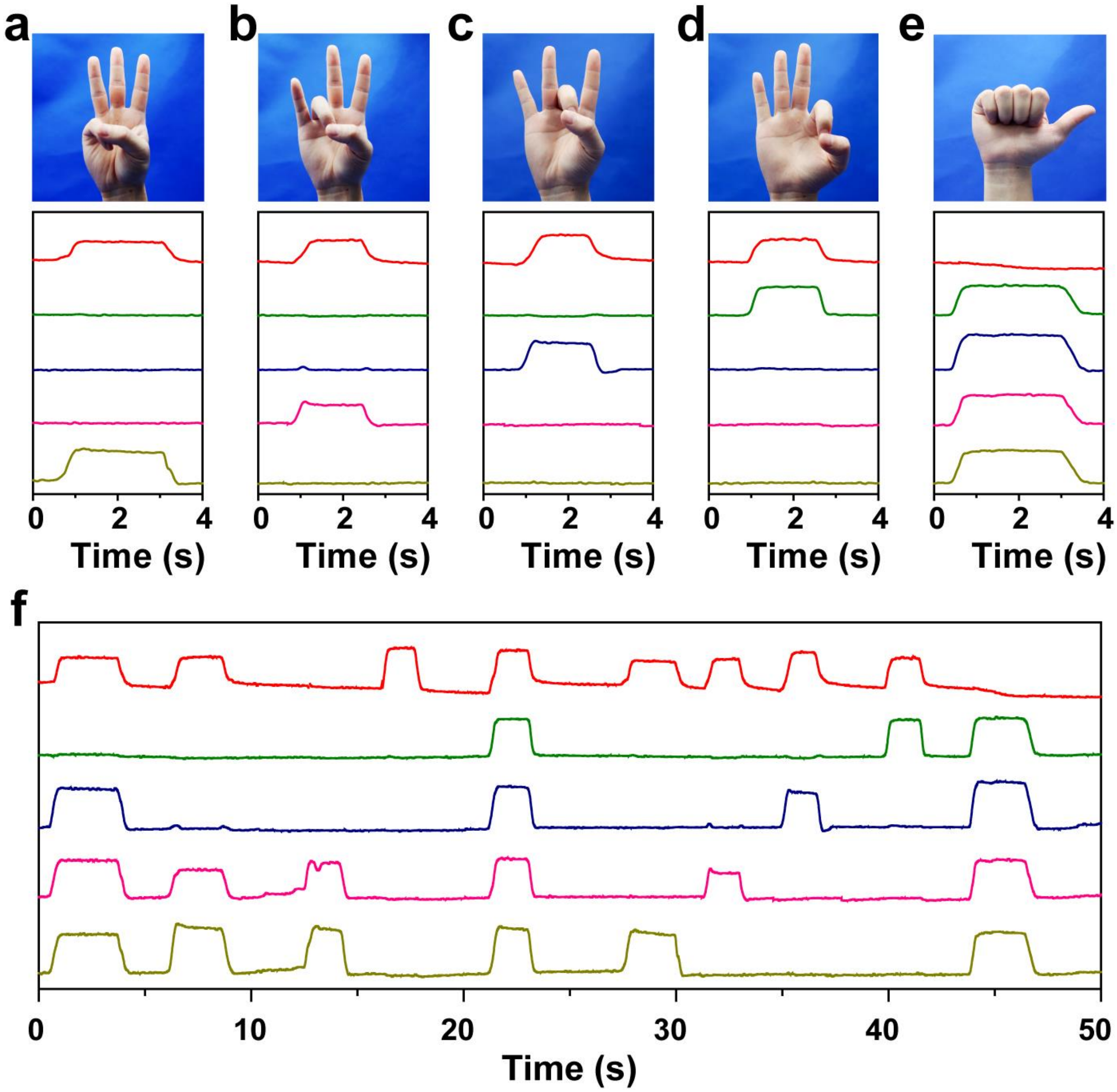

Figure S14. PWKCNT hydrogel modules for gesture-to-recognition translation. a-e) Photographs and electrical signals corresponding to the sign language numbers 6, 7, 8, 9, and 10, respectively. f) Electrical signals corresponding to the sign language numbers 1-10.

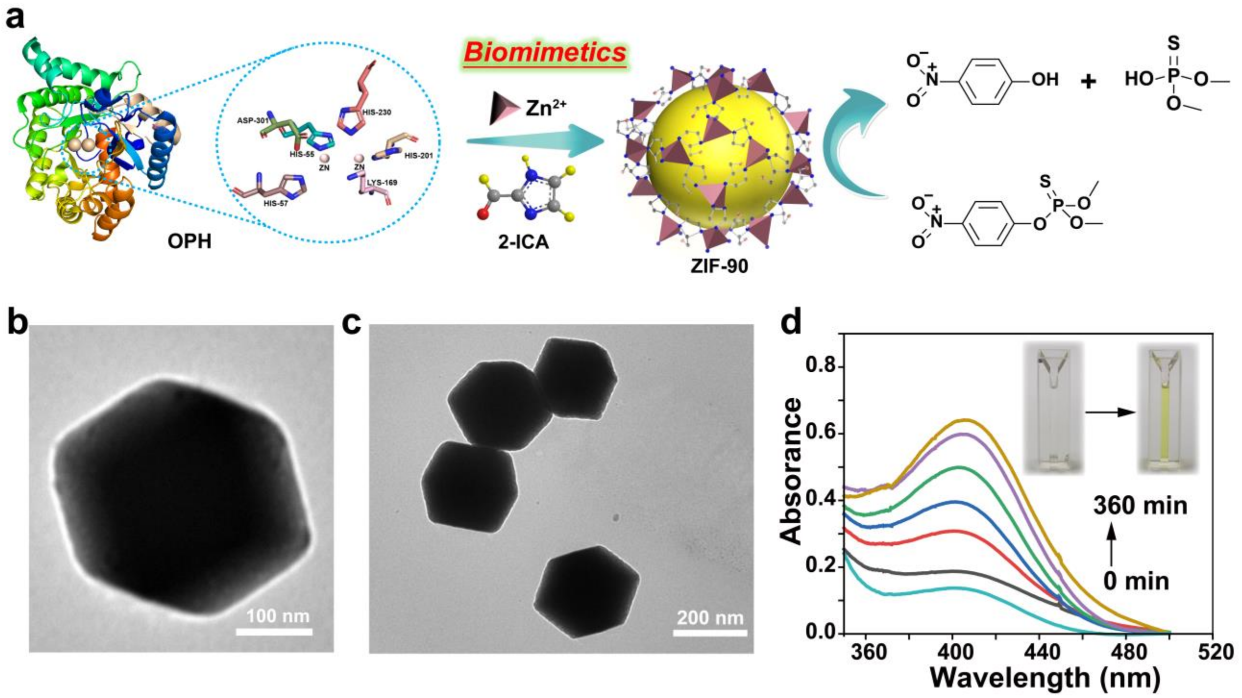

Figure S15. a) Schematic illustration of the synthesis and catalytic mechanism of nanozyme ZIF-90. b) TEM image of ZIF-90 nanozyme synthesized by the robot. c) TEM image of ZIF-90 nanozyme synthesized by a human. d) Absorbance spectra of the product p-nitrophenolate over time in the presence of ZIF-90 synthesized by the robot.

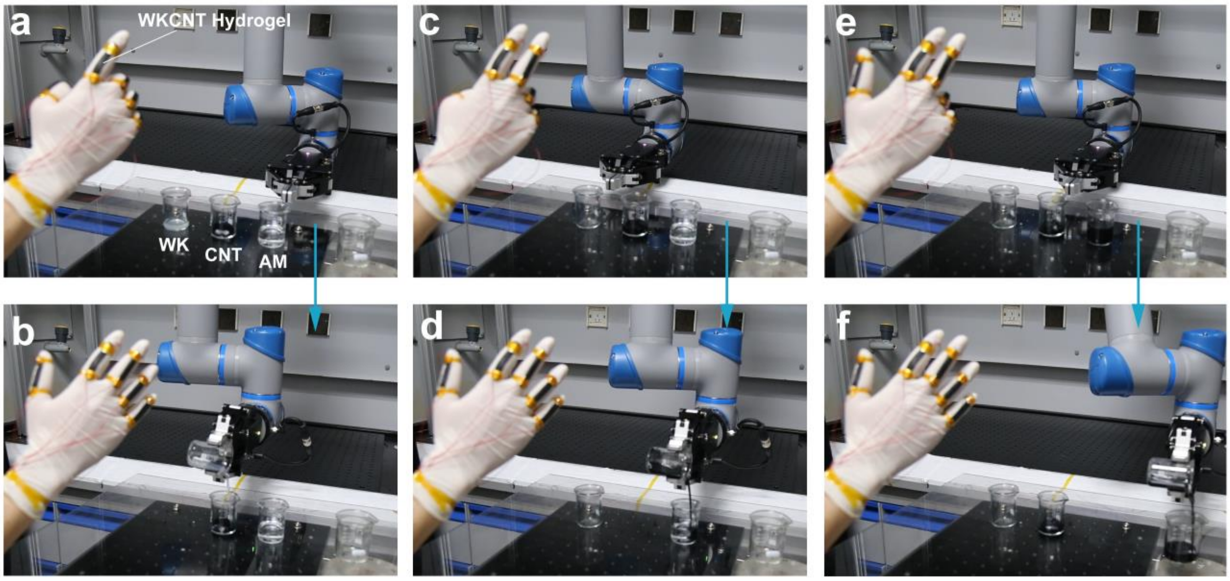

Figure S16. Fabrication of PWKCNT hydrogels based on PWKCNT sensing units. a-b) WK dispersed CNTs to obtain WKCNT bio-ink. c-d) WKCNT bio-ink was blended with a pre-prepared acrylamide solution. e-f)

The mixture was transferred to a beaker containing the catalyst and stirred until it became gelatinized.

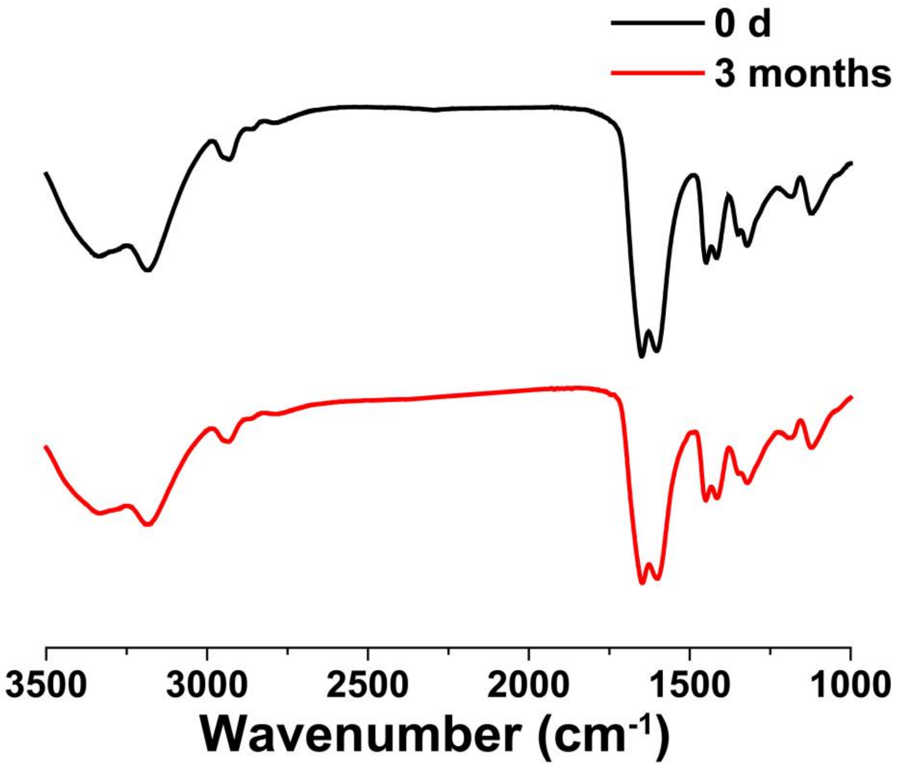

Figure S17. FTIR spectra of freshly made PWKCNT hydrogel and tested PWKCNT hydrogel left for three months.

[1] Y. Y. Diao, X. Y. Liu, G. W. Toh, L. Shi, J. Zi, *Adv. Funct. Mater.*, **2013,** *23*, 5373.
[2] S. Zhu, W. Zeng, Z. Meng, W. Luo, L. Ma, Y. Li, C. Lin, Q. Huang, Y. Lin, X. Y. Liu, *Adv. Mater.*, **2019,** *31*, 1900870.
[3] X. Liang, H. Li, J. Dou, Q. Wang, W. He, C. Wang, D. Li, J. M. Lin, Y. Zhang, *Adv. Mater.*, **2020,** *32*, 2000165.
[4] M. Lin, Z. Zheng, L. Yang, M. Luo, L. Fu, B. Lin, C. Xu, *Adv. Mater.*, **2021,** *34*, 2107309.
[5] C. H. Lee, M. S. Kim, B. M. Chung, D. J. Leahy, P. A. Coulombe, *Nat. Struct. Mol. Biol.*, **2012,** *19*, 707.
[6] D. C. Wang, H. Y. Yu, L. Jiang, D. Qi, X. Zhang, L. Chen, W. Lv, W. Xu, K. C. Tam, *Nano. Res.*, **2022,** *15*, 2616.
[7] Y. Zhang, C. Chen, Y. Qiu, L. Ma, W. Qiu, R. Yu, W. Yu, X. Y. Liu, *Adv. Funct. Mater.*, **2021,** *31*, 2100150.
[8] M. Amjadi, M. Sitti, *Adv. Sci.*, **2018,** *5*, 1800239.
